# Stability of transcranial magnetic stimulation electroencephalogram evoked potentials in pediatric epilepsy

**DOI:** 10.1038/s41598-024-59468-8

**Published:** 2024-04-20

**Authors:** Xiwei She, Kerry C. Nix, Christopher C. Cline, Wendy Qi, Sergei Tugin, Zihuai He, Fiona M. Baumer

**Affiliations:** 1 Department of Neurology, Stanford University, Stanford, CA USA; 2 Department of Psychiatry and Behavioral Sciences, Stanford University, Stanford, CA USA

**Keywords:** Epilepsy, Biomedical engineering, Computational neuroscience

## Abstract

Transcranial magnetic stimulation paired with electroencephalography (TMS–EEG) can measure local excitability and functional connectivity. To address trial-to-trial variability, responses to multiple TMS pulses are recorded to obtain an average TMS evoked potential (TEP). Balancing adequate data acquisition to establish stable TEPs with feasible experimental duration is critical when applying TMS–EEG to clinical populations. Here we aim to investigate the minimum number of pulses (MNP) required to achieve stable TEPs in children with epilepsy. Eighteen children with Self-Limited Epilepsy with Centrotemporal Spikes, a common epilepsy arising from the motor cortices, underwent multiple 100-pulse blocks of TMS to both motor cortices over two days. TMS was applied at 120% of resting motor threshold (rMT) up to a maximum of 100% maximum stimulator output. The average of all 100 pulses was used as a “gold-standard” TEP to which we compared “candidate” TEPs obtained by averaging subsets of pulses. We defined TEP stability as the MNP needed to achieve a concordance correlation coefficient of 80% between the candidate and “gold-standard” TEP. We additionally assessed whether experimental or clinical factors affected TEP stability. Results show that stable TEPs can be derived from fewer than 100 pulses, a number typically used for designing TMS-EEG experiments. The early segment (15–80 ms) of the TEP was less stable than the later segment (80–350 ms). Global mean field amplitude derived from all channels was less stable than local TEP derived from channels overlying the stimulated site. TEP stability did not differ depending on stimulated hemisphere, block order, or antiseizure medication use, but was greater in older children. Stimulation administered with an intensity above the rMT yielded more stable local TEPs. Studies of TMS-EEG in pediatrics have been limited by the complexity of experimental set-up and time course. This study serves as a critical starting point, demonstrating the feasibility of designing efficient TMS–EEG studies that use a relatively small number of pulses to study pediatric epilepsy and potentially other pediatric groups.

## Introduction

Transcranial magnetic stimulation paired with electroencephalography (TMS–EEG) provides a non-invasive way to investigate cortical excitability and functional connectivity. TMS evoked potentials (TEPs) are waveforms with characteristic peaks reflecting local and long-range excitatory or inhibitory responses to stimulation of the cortex^1,2^. TEPs have shed light on the function of brain networks during behavioral tasks^3^, the pathophysiology of disease^4^, and the pharmacodynamics of neuroactive medications including antiseizure medications (ASMs)^5,6^.

TMS–EEG experiment sessions are often lengthy, making them challenging for many clinical populations including children. One factor contributing to study length is that typically 100–200 pulses are collected and averaged to derive a TEP for each condition or cortical region of interest^7–9^. Enough pulses must be applied such that, when averaged, the signal of interest (the TEP) is distinct from the ongoing, background brain activity as well as artifacts (noise). Here, we define a “stable” TEP as one that does not change with additional pulses and aim to quantify the minimum number of pulses (MNP) required to achieve this stability. MNP depends on the signal-to-noise ratio (SNR), which can be affected by experimental factors (e.g., stimulation intensity^10,11^, stimulation duration^12^, and coil orientation^13^) and biological factors (e.g., age^14^, gender^15^, and genetics^16^). Additionally, certain stimulation sites are less prone to artifact; for example, a stable primary motor cortex TEP can be derived with less than 100 pulses^17–19^, whereas a parietal cortex TEP may require 130–180 pulses^20–22^. An understanding of the MNP enhances efficiency, allowing for shorter studies or studies in which researchers can explore a wider array of conditions within a single experimental day. The SNR may also be enhanced by shorter study protocols as there will be fewer artifacts related to participant fatigue. Moreover, studies requiring high temporal precision (e.g., plasticity studies or studies of temporally specific phenomenon like seizures) benefit from shorter assessment blocks. Therefore, understanding the MNP needed for a given population is particularly meaningful for efficient design of clinical TMS–EEG studies.

TMS–EEG studies in children^23,24^ have generally used a similar number of pulses as are used in adult experiments, but to our knowledge, the MNP required in children has not been explicitly explored. Children have higher amplitude evoked potentials^25,26^ but may be less cooperative, thus raising the question as to whether their TEPs are more or less stable than those of adults. Here, in the context of an ongoing clinical trial (NCT04325282), we assessed the MNP required to achieve stable TEPs from the motor cortex in a group of children with self-limited epilepsy with centrotemporal spikes (SeLECTS). SeLECTS is a common pediatric focal epilepsy syndrome, in which children have macroscopically normal brain anatomy but develop seizures originating in one or both sensorimotor cortices. Seizures are rare and occur almost entirely during sleep. About half of children do not take daily ASMs. Children with SeLECTS therefore represent a fairly homogenous group^27^ for assessing the MNP. We assessed the stability of both the local TEP and global mean field amplitude (GMFA) responses in the early (15–80 ms) and late (80–350 ms) response period. Moreover, we tested whether experimental or clinical factors affected the stability of the TEP.

## Methods

### Participants

Right-handed children aged 7–13 years with SeLECTS were recruited from Lucile Packard Children’s Hospital. Children had a history of at least one focal motor seizure and an EEG with sleep-potentiated spikes predominantly in a centrotemporal distribution. Exclusion criteria included a history of a severe neurologic disorder (e.g., neonatal encephalopathy, stroke), focal neurologic deficits, or prematurity. Imaging was not a prerequisite as it is normal in children with SeLECTS, but children with abnormal imaging identified as part of clinical care were excluded. The study was approved by the Stanford University Institutional Review Board. Written informed consent was obtained from parents and assent from children. Medication use was recorded. All experiments were performed in accordance with relevant guidelines and regulations.

### Experimental set-up

EEG recordings were obtained with a 64-channel ActiCAP slim active electrodes and BrainVision ActiCHamp Plus amplifier, sampling at 25 kHz. TMS was administered using a Magventure X100 stimulator via a Cool-B65 figure-8 coil, guided by the Localite TMS Navigator system for neuro-navigation, registered either to the patient’s T1-weighted anatomical magnetic resonance image (MRI) or to a representative MRI from unbiased average age-appropriate templates^28^. Electromyographic (EMG) recordings were measured from the bilateral abductor pollicis brevis (ABP) muscles, sampled at 25 kHz and high pass filtered at 1 Hz.

All sessions occurred in the late morning or early afternoon. Participants were seated comfortably in a semi-reclined position, and underwent TMS to the motor cortices. Impedances were kept below 10 kΩ; though impedances < 5 kΩ are preferred^10^, this higher threshold was used to ensure that set-up time was feasible for children. After cap application, we identified the motor “hotspot” of each hemisphere as the cortical location that, when stimulated, elicited the largest EMG deflection in the ABP EMG electrode when the hand was relaxed. If no EMG deflection was observed at maximal stimulator output (MSO), we repeated motor hotspot identification with the hand slightly contracted. The resting motor threshold (rMT) of each motor cortex was defined as the minimum intensity to evoke a peak-to-peak EMG signal of at least 50 µV in at least 5 out of 10 pulses^29^. In participants whose rMT exceeded 100% MSO, we defined rMT as 100% MSO. We did not attempt to measure an active motor threshold, because our pediatric patients could not reliably exert a specific amount of pressure, making aMT highly inconsistent. The sound of the TMS click was masked with in-ear headphones playing white noise, with a frequency matched with the frequency of TMS clicks^30^ and with a volume in which children reported difficulty hearing the click but no discomfort. A foam layer was applied to the coil to reduce the vibration from the TMS. Alertness was confirmed and maintained during the whole session via observation of the participant and EEG signals.

### Stimulation blocks

**Figure 1:**
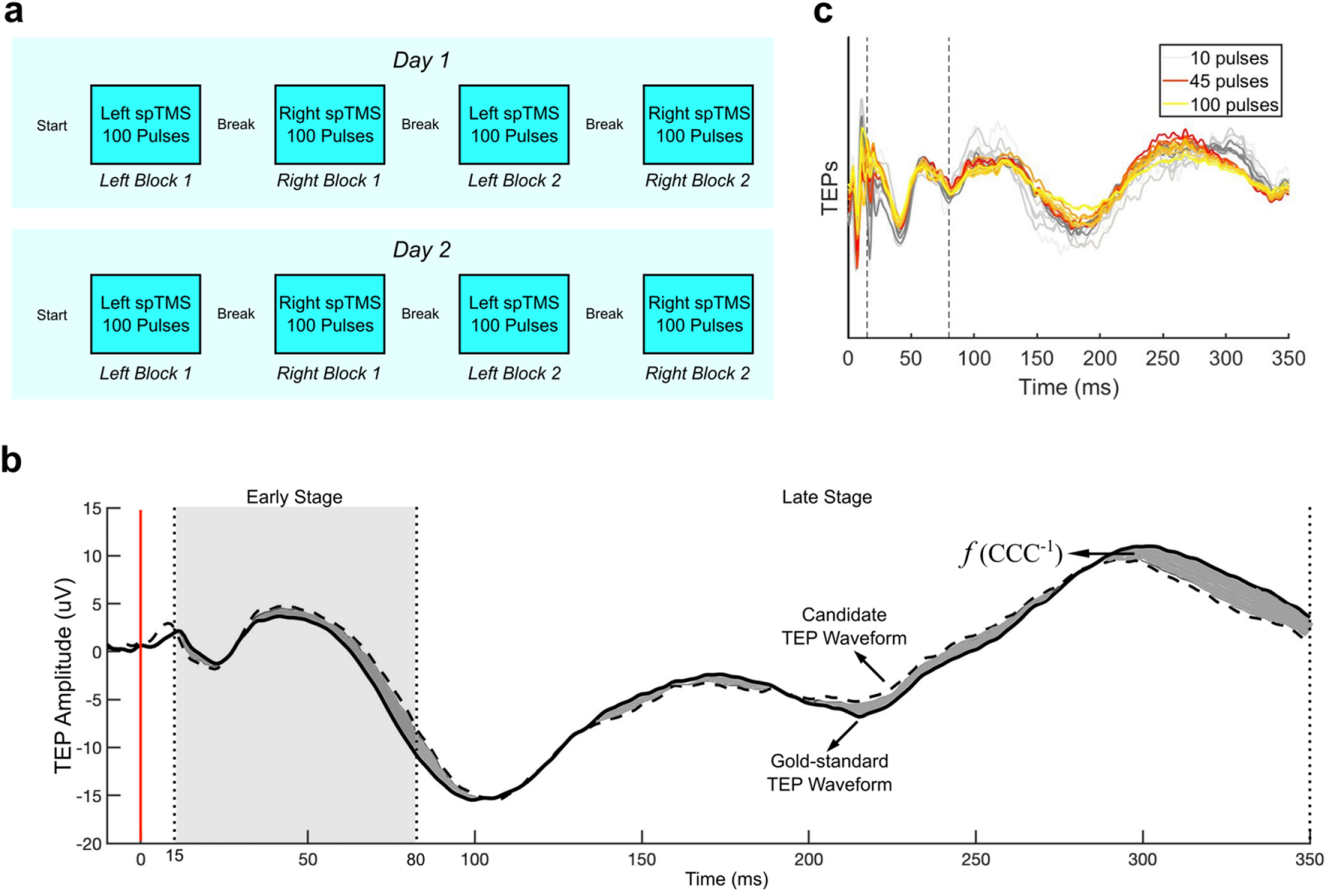
Experimental set-up and TMS-evoked potential (TEP) stability analyses. (**a**) Example layout of experiment for Day 1 and Day 2, showing 4 100-pulse blocks alternating between the two motor cortices. Initial hemisphere stimulated was randomized across participants; (**b**) TEP stability was quantified by calculating the concordance correlation coefficients (CCC) between the candidate TEP waveform (dashed gray curve) and the gold standard TEP waveform (black solid curve). The candidate TEP waveform was derived using the first n pulses and the gold standard waveform was derived using all pulses. The CCC is proportional to the reciprocal of the area between the two waveforms (gray area). (**c**) Example data showing that with 45 or more TMS pulses, the candidate TEP waveform (red curve) achieved high concordance with the gold standard TEP waveform (yellow curve). The CCC was 0.89 and 0.92 for the early and late segment, respectively.

Participants underwent four 100-pulse blocks (2 left, 2 right) of TMS, alternating between the motor hotspot of each hemisphere. The initial stimulation side was randomized on a per-participant basis. Participants returned for a second day of stimulation at least 6 days after the first session, and thus underwent up to 8 blocks. rMT was confirmed again on the second day. Stimuli were administered at 120% rMT (or 100% MSO for those with rMT ≥ 84% MSO), with pulses jittered at random intervals between 2 and 3 s. Short 1 to 3-min breaks were given between blocks (Fig. 1a). Several children were unable to tolerate four blocks on each day, so we included all available data for each participant.

### Stability criteria

We first defined the “gold-standard” TEP waveform for each block by preprocessing all 100 pulses within each block together. Subsequently, we conducted a progressive preprocessing approach on a subset of pulses, beginning with including only 10 pulses and iteratively adding 5 consecutive pulses at each step. This allowed us to calculate candidate TEP waveforms at various steps of pulse inclusion. We compared each candidate waveform to the gold-standard waveform using the concordance correlation coefficient (CCC)^31^, a measurement of similarity that is proportional to the reciprocal of the area between the two waveforms (Fig. 1b). The MNP was determined after the threshold CCC of 0.8^32,33^ was surpassed (Fig. 1c). We confirmed that the MNP was not a “local minimum” by testing all subsequent candidate TEPs (calculated by adding more pulses) to ensure that they also had a CCC > 0.8.

### TEP and GMFA

We assessed the stability of both the local TEP and GMFA responses. Local TEPs were derived from 5 electrodes overlying the site of stimulation (channels C3, C1, C5, FC3, and CP3 for left motor cortex stimulation; channels C4, C2, C6, FC4, and CP4 for right motor cortex stimulation). GMFA was derived from all electrodes. We looked at the local TEP and GMFA as these are two typical measures providing insights into the temporal dynamics, spatial distributions, and overall responses magnitude to the TMS-induced brain activity^34^.

### Time window

We calculated CCC across two time-windows, the early waveform (15–80 ms), and the late waveform (80–350 ms). We looked at the early waveform as these peaks reflect local cortical responses^35,36^. We also examined the later waveform, because pediatric data is dominated by large, lateralized peaks, particularly an N100, and simple early waveforms, with few well-defined peaks before 50–60 ms^24^, differing from typical TEPs seen in adults^37^.

### Data preprocessing

We preprocessed TMS–EEG data with EEGLAB^38^ running in the MATLAB environment (version 2022a). We preprocessed all 100 pulses together to derive the gold-standard TEP. We then preprocessed the subset of pulses for each candidate TEP separately, mimicking an experiment in which we had collected fewer pulses. For each preprocessing batch, the data was first epoched within a time window spanning from − 1000 to 1500 ms around the TMS pulse. The following preprocessing steps were adapted from the AARATEP pipeline^39,40^, with a summary of relevant procedures given here. To eliminate the primary TMS pulse artifact, an interpolation procedure was applied within a time window spanning from − 2 to 12 ms relative to the pulse onset. This interpolation step entailed fitting autoregressive models to 20 ms of data both before and after the interpolation timespan^40^. Subsequently, the data was down-sampled to 1 kHz and baseline corrected by subtracting the mean within the − 500 to − 10 ms timespan from all data points. A high-pass filter was then applied to the data to retain frequencies above 1 Hz, with piecewise extrapolation and filtering to minimize convolutional spreading of post-stimulation response into the pre-stimulation time period. Rejection of bad channels was accomplished using a data-driven Wiener noise estimation method^41^. Further noise reduction was achieved by using the SOUND algorithm^42^ using a lambda regularization parameter of 10^−1.5^. Line noise was attenuated by a Butterworth bandstop filter (58–62 Hz). Independent component analysis with classification via the ICLabel algorithm^43^ was then used to identify and further remove artifacts. Following the ICLabel step, components were also screened for remaining artifacts during the 11–30 ms time window (where TMS-induced muscle artifacts commonly occur). This rejection was inspired by a similar rule from the TESA toolbox^44,45^. The final preprocessed signals from all channels were obtained after low pass filtering below 200 Hz and re-referencing to a common average.

For each participant’s full dataset (i.e., preprocessed using all 100 pulses), trials with large movement or muscle artifacts were rejected by visual inspection performed by a research assistant (WQ) and confirmed by a board-certified epileptologist (FB) using EEGLAB. The number of rejected trials for each participant were recorded. We compared the final TEPs with versus without the bad trial rejection for all participants to test if bad trials affect the stability in our cohort.

### Impact of experimental and clinical factors on TEP stability

We investigated the impact of three experimental factors (hemisphere, block order, and day of stimulation) and three clinical factors (rMT, ASM use, and age) on TEP stability. We did not examine the impact of sex on TEP stability as the epilepsy under investigation skews male.

*Hemisphere stimulated*: We tested whether TEPs were more stable in the left or right motor cortex. All participants were right-handed.

*Block & day order*: We tested if TEP stability differed based on block order within a day, defining “block one” as the first block administered regardless of hemisphere. For participants who underwent 2 days of stimulation, we tested if the day of TMS influenced stability.

*Stimulation intensity relative to rMT:* TEP shape and amplitude vary with stimulation intensity^10,11^. Stimulation as low as 60% rMT can elicit TEPs^11^, but studies typically stimulate above rMT to improve SNR. Children have higher rMTs than adults^46^, sometimes exceeding MSO. We modeled stimulation intensity relative to rMT as a binary variable. Since TMS was applied at 120% of rMT up to a maximum of 100% MSO, participants with an rMT ≥ 100% MSO were classified as having received **subthreshold stimulation** and participants with rMT < 100% MSO were classified as having received **suprathreshold stimulation**.

*ASM use:* Half of children with SeLECTS take daily ASMs^23^ (typically oxcarbazepine or levetiracetam in our practice). ASMs impact cortical excitability and TEP amplitude^5,47^. We defined ASM use as a binary variable; all children on ASMs had a stable dose for at least one month prior to the study.

*Age:* We modeled age as a continuous variable. Given that younger children may be less able to tolerate TMS–EEG experiments, we additionally quantified the number of channels, percentage of variance in rejected components, and number of trials rejected by preprocessing steps as a function of age to estimate if more pulses are required in younger children for this reason.

### Statistical analysis

Statistics were computed using Statistical Analysis System (SAS) OnDemand for Academics^48^. Since subjects contributed multiple MNP outcome measurements to each model, we used a generalized estimating equation (GEE) with an exchangeable correlation matrix^49^ to account for repeated-measures and correlation within individuals for each of the analyses. We first conducted univariate analyses to determine if MNP differed based on stimulation site, day, block order, stimulation intensity relative to rMT, ASM use, or age. We also performed a GEE model to test if age is significantly associated with the number of rejected channels, percentage of variance in rejected components, or number of rejected trials. Furthermore, as both age^14^ and ASM use^47^ are associated with elevated rMT, we performed a multivariable analysis to test the effect of each of these factors on MNP when adjusting for the others.

### Comparison of preprocessing pipelines (Appendix A)

Different preprocessing pipelines can yield different outcomes^10,45^, and thus a growing recommendation is to use “multiverse analyses”^50^, i.e., preprocess data using more than one pipeline. Therefore, as a supplementary analysis, we additionally pre-processed 12 blocks from 3 age-matched subjects using the TESA pipeline^44,45^, a common method for TMS-EEG data preprocessing that can be run either semi- or fully-automatically. In this supplementary comparison, we used the fully-automatic version (without manual bad trial rejection). We compared the early and late segments of TEP and GMFA derived from two pipelines using CCC. We also compared the MNP derived from two pipelines using paired t-tests. Please see Supplementary Materials (Appendix A) for further details.

### Comparison of pulse inclusion methods (Appendix B)

To address the potential risk of entrainment in which TEPs are influenced by exposure to previous pulses, we conducted an additional analysis comparing MNP when consecutive pulses were included vs. MNP when pulses were randomly included regardless of the temporal order that they were administered. We selected 2 blocks of TMS data from each of the 18 subjects, resulting in a total of 36 blocks for analysis. This approach aimed to minimize any potential order effects or biases from consecutive pulse inclusion. Please see Supplementary Materials (Appendix B) for further details.

### Ethical approval

The study was approved by the Stanford University IRB with waiver of patient consent given the retrospective nature of the research.

## Results

### Participants

Eighteen right-handed children with SeLECTS, ranging from 7 to 13 years (mean 10.1 + / − 1.5) were included. Eight (44.4%) took ASMs (four levetiracetam and four oxcarbazepine). Children had high rMT in the left (85.8 + / − 14.5% MSO) and right (89.0 + / − 13.2% MSO) hemispheres. Six children had rMT exceeding 100% MSO bilaterally, nine children had rMT lower than 100% MSO bilaterally, and three children had rMT lower than 100% MSO in one but higher than 100% MSO in the other hemisphere. Three participants came for a single day and fifteen completed two days. rMT was stable across days with no more than a 2% variance. In total, 122 100-pulse blocks were completed.

For all participants, an average of 1.2 + / − 0.2 channels were rejected by the preprocessing step, and an average of 49.5% + / − 10.5% of components were rejected, accounting for 30.2% + / − 16.9% of variance in the signals at this stage. We manually inspected the final “gold standard” blocks of 100 trials and identified that an average of 4.0 + / − 2.1 trials were contaminated with large movement artifacts. Nevertheless, the final TEP with bad trial rejection had a CCC > 0.8 to the final TEP without bad trial rejection in all 122 blocks.

The average and the standard deviation of MNP reported in this paper have been rounded to the nearest whole number.

### TEP stability (Fig. 2)

**Figure 2:**
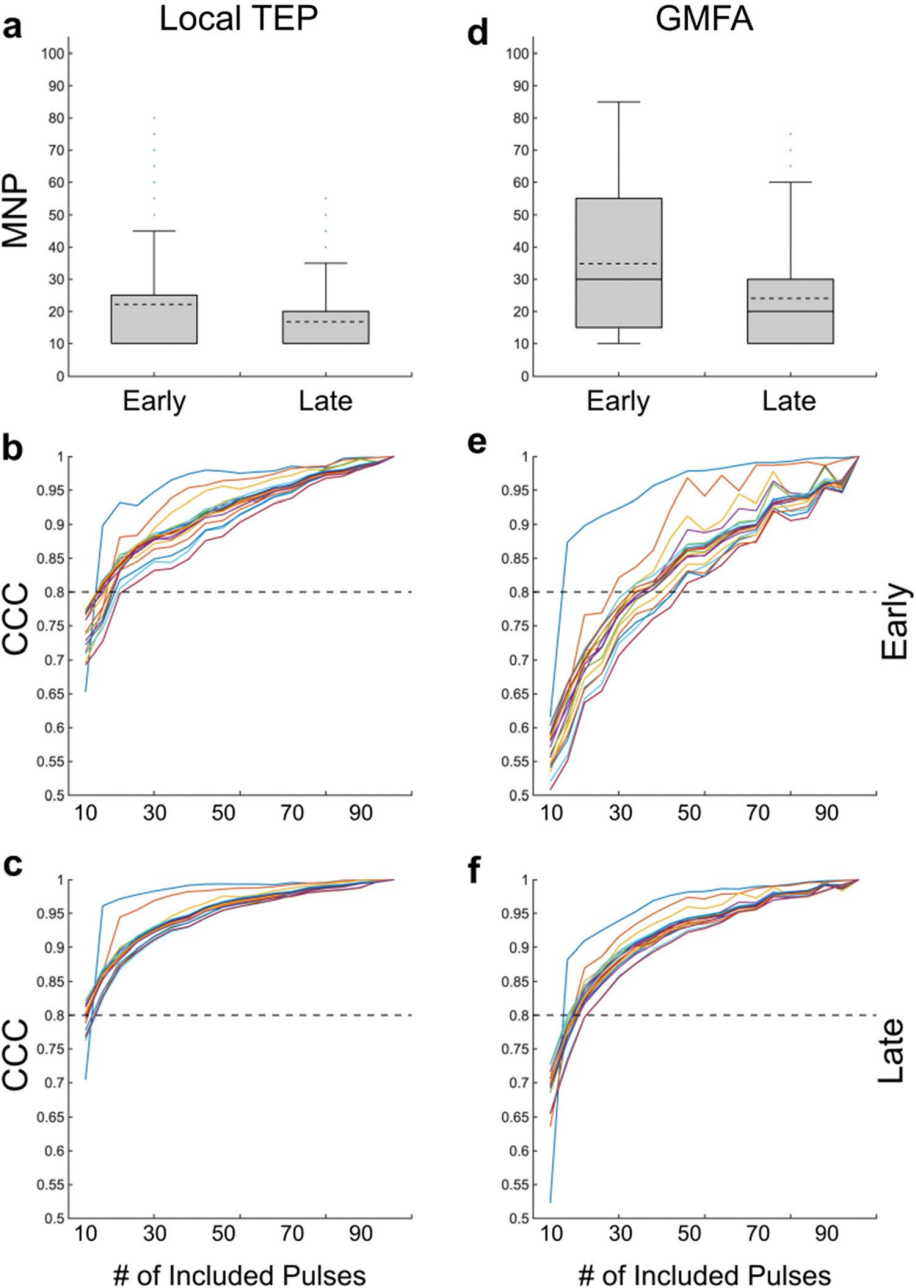
TEP stability in children with epilepsy. The left column (**a**, **b**, **c**) depicts stability of the local TMS-evoked potential (TEP) while the right column (**d**, **e**, **f**) depicts stability of the global mean field amplitude (GMFA). Minimal number of pulses (MNP) required to achieve stable early (15–80 ms) and late (80–350 ms) (**a**) local TEP and (**d**) GMFA waveforms. Box plots show mean (dashed line), median (solid line), interquartile range (shaded region), outliers (dots) which MNP are higher than 99% coverage of the data (whiskers). Subject-level data show an increase in concordance correlation coefficient (CCC) between the candidate waveforms and the gold standard waveform as number of pulses increases for: (**b**) the early local TEP; (**c**) the late local TEP; (**e**) the early GMFA; and (**f**) the late GMFA. Each colored line represents one participant’s results averaged across blocks.

*Early Waveform (15–80 ms):* The early segment of the local TEP required an average of 22 + /- 19 pulses to reach stability. Eighty percent of blocks (99/122, 81.2%) reached stability with 25 pulses and all blocks reached stability by 80 pulses.

Compared to the local TEP, more pulses were required to achieve stability of the early segment of the GMFA, requiring an average of 35 + / − 22 pulses. Eighty percent of blocks (103/122, 84.4%) reached stability with 60 pulses and all blocks reached stability by 85 pulses.

*Late Waveform (80–350 ms):* The late segment of the local TEP required an average of 17 + / 11 pulses to reach stability. Eighty percent of blocks (102/122, 83.6%) reached stability with 20 pulses and all blocks reached stability by 55 pulses.

To achieve stability of the late segment of the GMFA, an average of 24 + / − 17 pulses were required. Eighty percent of blocks (101/122, 82.8%) reached stability with 30 pulses, and all blocks reached stability by 75 pulses.

### Effect of experimental & clinical factors on stability (Tables 1, 2)

**Table 1:** Impact of Experimental & Clinical Factors on local TEP stability.

	Early (15–80 ms)			Late (80–350 ms)		
Factor of interest	Estimate (CI)	Z	*p*-value	Estimate (CI)	Z	*p*-value
Experimental factors						
Hemisphere		1.4	0.16		− 0.3	0.79
Left	20.1 (3.9, 36.2)			17.0 (5.2, 28.8)		
Right	25.1 (2.0, 48.2)			16.4 (1.0, 32.7)		
Block order						
(Blocks 2–4 vs. Block 1)						
Block 1	20.5 (13.6, 27.4)			16.4 (12.4, 20.5)		
Block 2	22.2 (8.1, 36.3)	0.5	0.65	15.2 (5.4, 25.0)	− 0.4	0.68
Block 3	26.0 (13.3, 38.7)	1.9	0.06	17.8 (9.2, 26.4)	0.6	0.56
Block 4	22.1 (7.1, 37.1)	0.4	0.71	17.6 (7.6, 27.6)	0.4	0.69
Day		**2.6**	**0.01**		1.8	0.07
Day 1	16.6 (0.2, 33.0)			14.6 (5.2, 24.0)		
Day 2	26.7 (2.7, 50.7)			18.2 (4.9, 31.5)		
Clinical factors						
Stim intensity relative to rMT		** − 2.3**	**0.02**		− 1.8	0.07
Supra	18.2 (0.5, 35.8)			15.2 (8.3, 22.0)		
Sub	28.4 (19.6, 37.2)			18.9 (16.0, 21.7)		
ASM use		− 0.2	0.82		1.8	0.08
Yes	21.8 (1.9, 41.7)			19.2 (12.1, 26.3)		
No	23.1 (14.6, 31.6)			15.0 (12.6, 17.4)		
Age		** − 3.5**	** < 0.001**		** − 2.7**	**0.006**
MNP at 7 years	36.9 (9.2, 82.9)			21.6 (1.5, 41.7)		
Change/year	− 4.6 (− 7.2, − 2.1)			− 1.6 (− 2.7, − 0.4)		

ASM = Antiseizure medication; MNP = Minimum number of pulses; rMT = resting motor threshold; Stim = stimulation; Sub = subthreshold stimulation with intensity below the resting motor threshold; Supra = suprathreshold stimulation with intensity above the resting motor threshold.

Significant values are in bold.

**Table 2:** Impact of experimental & clinical factors on GMFA stability.

	Early (15–80 ms)			Late (80–350 ms)		
Factor of interest	Estimate (CI)	Z	*p*-value	Estimate (CI)	Z	*p*-value
Experimental factors						
Hemisphere		0.9	0.36		1.9	0.06
Left	34.0 (13.1, 54.9)			22.1 (10.9, 33.4)		
Right	37.7 (8.8, 66.7)			26.8 (10.9, 42.8)		
Block order						
(Blocks 2–4 vs. Block 1)						
Block 1	36.8 (28.5, 45.1)			25.4 (20.5, 30.4)		
Block 2	37.3 (19.5, 55.0)	0.1	0.92	24.5 (11.6, 37.4)	− 0.2	0.82
Block 3	32.8 (19.3, 46.4)	− 1.5	0.14	22.8 (12.2, 33.4)	− 0.9	0.37
Block 4	35.7 (18.9, 52.4)	− 0.3	0.80	24.8 (13.8, 35.7)	− 0.2	0.83
Day		1.1	0.25		1.0	0.30
Day 1	32.6 (9.5, 55.7)			22.2 (3.4, 41.0)		
Day 2	38.1 (5.5, 70.7)			26.1 (0.1, 52.3)		
Clinical factors						
Stim intensity relative to rMT		− 0.6	0.52		− 1.4	0.16
Supra	34.2 (13.3, 55.1)			22.0 (6.5, 37.5)		
Sub	38.0 (28.5, 47.4)			27.8 (20.4, 35.2)		
ASM use		− 0.3	0.80		− 1.2	0.24
Yes	34.8 (11.5, 58.2)			21.5 (6.8, 36.2)		
No	36.6 (26.8, 46.4)			26.6 (20.4, 32.8)		
Age		** − 5.8**	** < 0.001**		** − 4.3**	** < 0.001**
MNP at 7 years	55.6 (18.6, 92.7)			35.6 (6.3, 65.0)		
Change/year	− 6.4 (− 8.6, − 4.3)			− 3.6 (− 5.2, − 2.0)		

ASM = Antiseizure medication; MNP = Minimum number of pulses; rMT = resting motor threshold; Stim = stimulation; Sub = subthreshold stimulation with intensity below the resting motor threshold; Supra = suprathreshold stimulation with intensity above the resting motor threshold.

Significant values are in bold.

**Figure 3:**
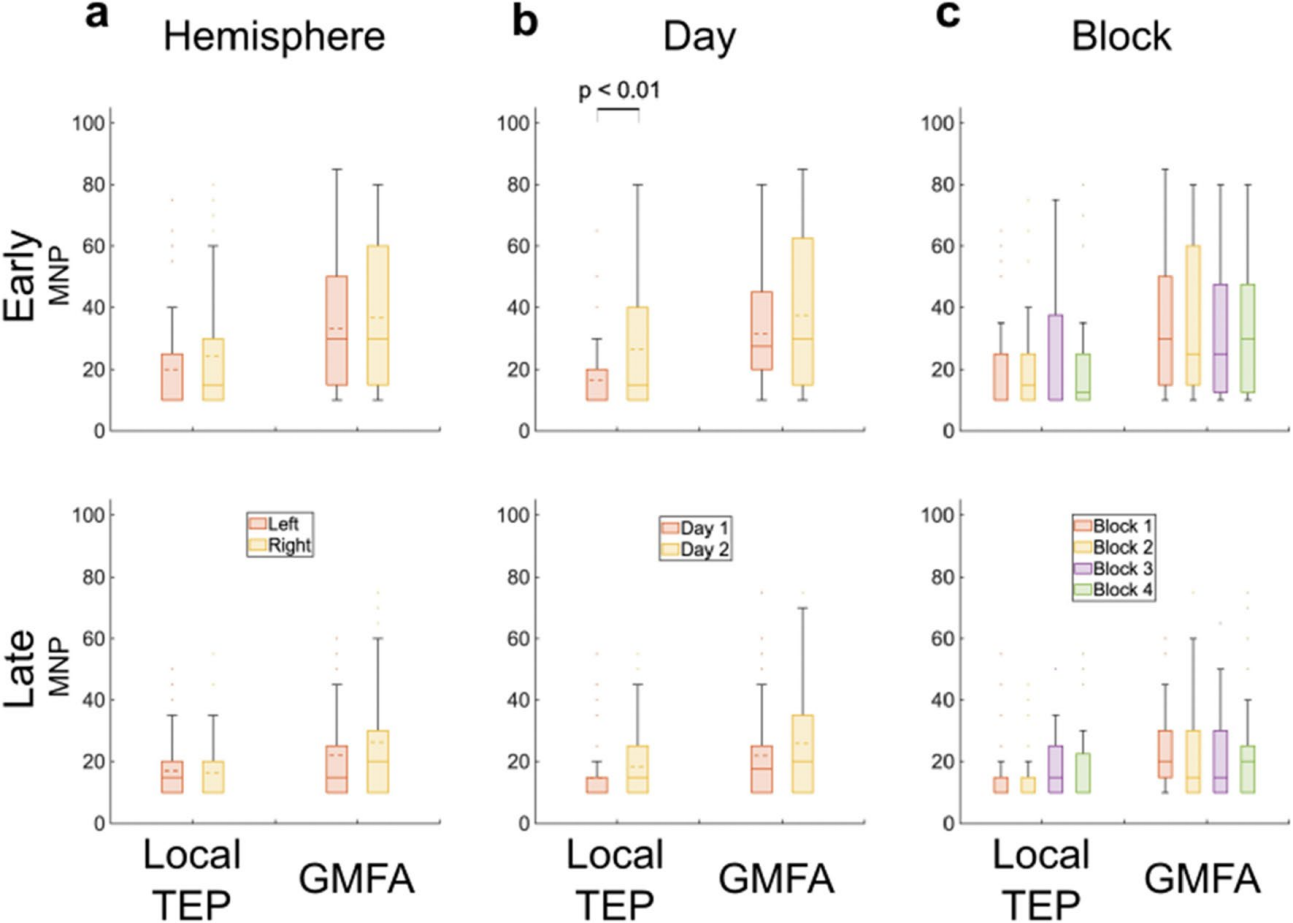
Impact of experimental factors on minimum number of pulses (MNP) necessary to achieve stability for the early (15–80 ms, top row) and late (80–350 ms, bottom row) waveforms. Box plots show mean (dashed line), median (solid line), interquartile range (shaded region), outliers (dots) which MNP are higher than 99% coverage of the data (whiskers). Impact of (**a**) stimulation site (left vs. right hemisphere); (**b**) stimulation day; and (**c**) block order on MNP.

*Experimental factors *(Fig. 3): TEP stability did not differ based on hemisphere stimulated or block order of stimulation. Stability differed between day 1 and 2 of stimulation only for the early segment of the local TEP; the MNP was lower on day 1 than day 2 (Day 1: 17 + / − 11; Day 2: 27 + / − 23; *p*-value = 0.01). The MNP did not differ across stimulation days for the late segment of the local TEP or for the GMFA.

**Figure 4:**
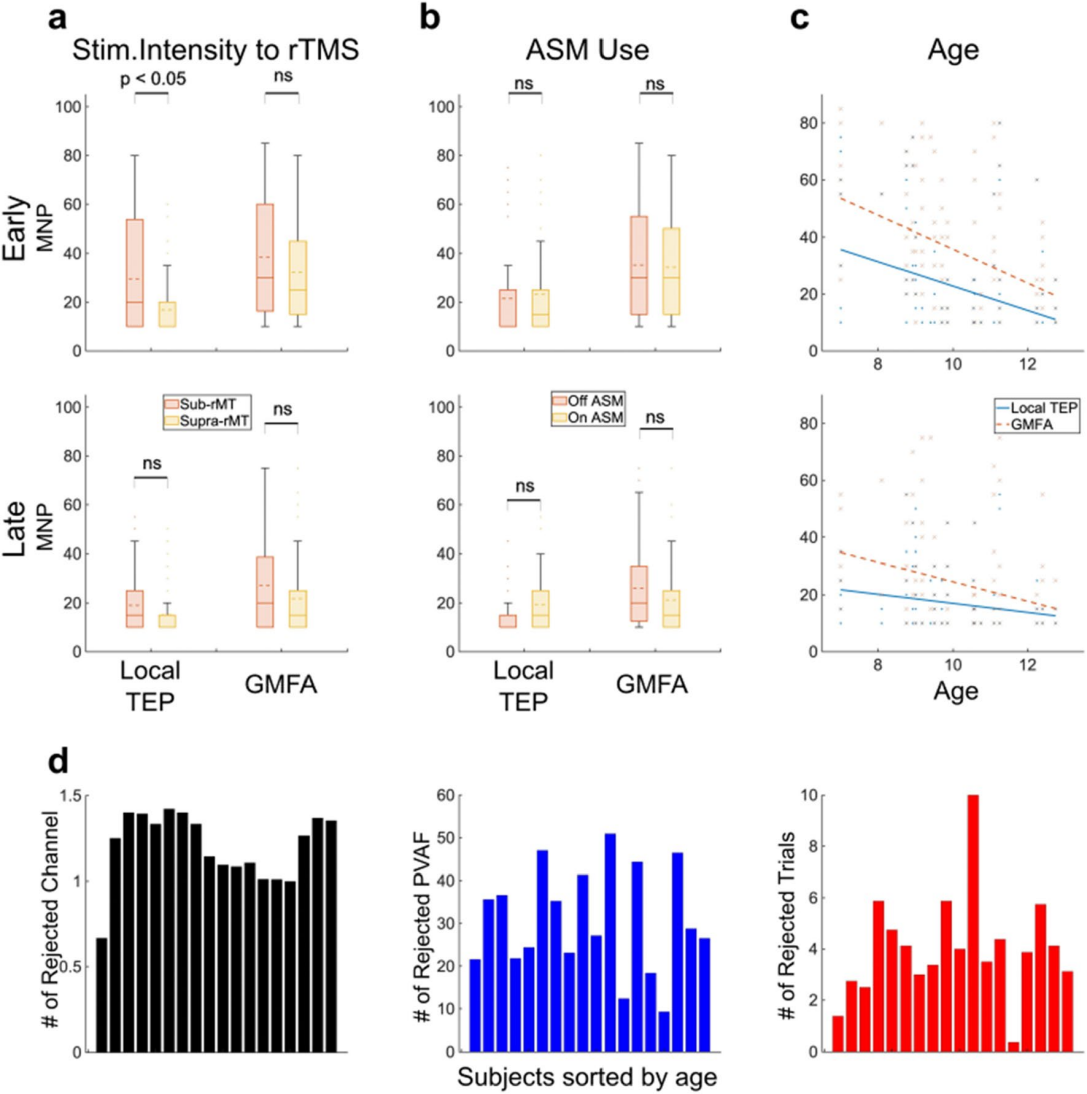
Impact of clinical factors on minimum number of pulses (MNP) necessary to achieve stability for the early (15–80 ms, top panels) and late (80–350 ms, middle panels) waveforms. Box plots show mean (dashed line), median (solid line), interquartile range (shaded region), outliers (dots) which MNP are higher than 99% coverage of the data (whiskers). (**a**) Impact of stimulation intensity relative to resting motor threshold (rMT; subthreshold stimulation with intensity below the rMT vs. suprathreshold stimulation with intensity above the rMT) on MNP. (**b**) Impact of antiseizure medication (ASM) use (On vs. Off) on MNP. (**c**) Impact of age on MNP. (**d**) The average number of channels (black), the average percentage of components’ variance (PVAF, blue), and the average number of trials (red) rejected during preprocessing steps across participants ordered by age.

*Stimulation intensity relative to rMT *(Fig. 4): Fewer pulses were required to reach stability in the early segment of local TEPs when the stimulation intensity used exceeded rMT (suprathreshold 17 + / − 12; subthreshold 30 + / − 24; Z =  − 2.26, *p*-value = 0.02). Stimulation intensity relative to rMT did not impact stability of the late segment of the local TEP or the GMFA in either the early or late segments.

*ASM use *(Fig. 4): Both local TEP and GMFA stability at the early and late segment did not differ significantly based on ASM use.

*Age *(Fig. 4): Stability significantly increased with age. For the local TEP, the MNP decreased by 5 pulses/year for the early (Z =  − 3.52, *p*-value = 0.0004), and by 2 pulses/year for the late (Z =  − 2.74, *p*-value = 0.006) waveforms. The MNP for GMFA decreased by 6 pulses/year for the early (Z =  − 5.80, *p*-value < 0.0001), and 4 pulses/year for the late (Z =  − 4.34, *p*-value < 0.0001) waveforms.

Data quality (i.e., the number of channels, components, or trials rejected by preprocessing steps) did not significantly change with age.

### Adjusted effect of clinical factors on TEP stability (Fig. 5, Table 3)

**Figure 5:**
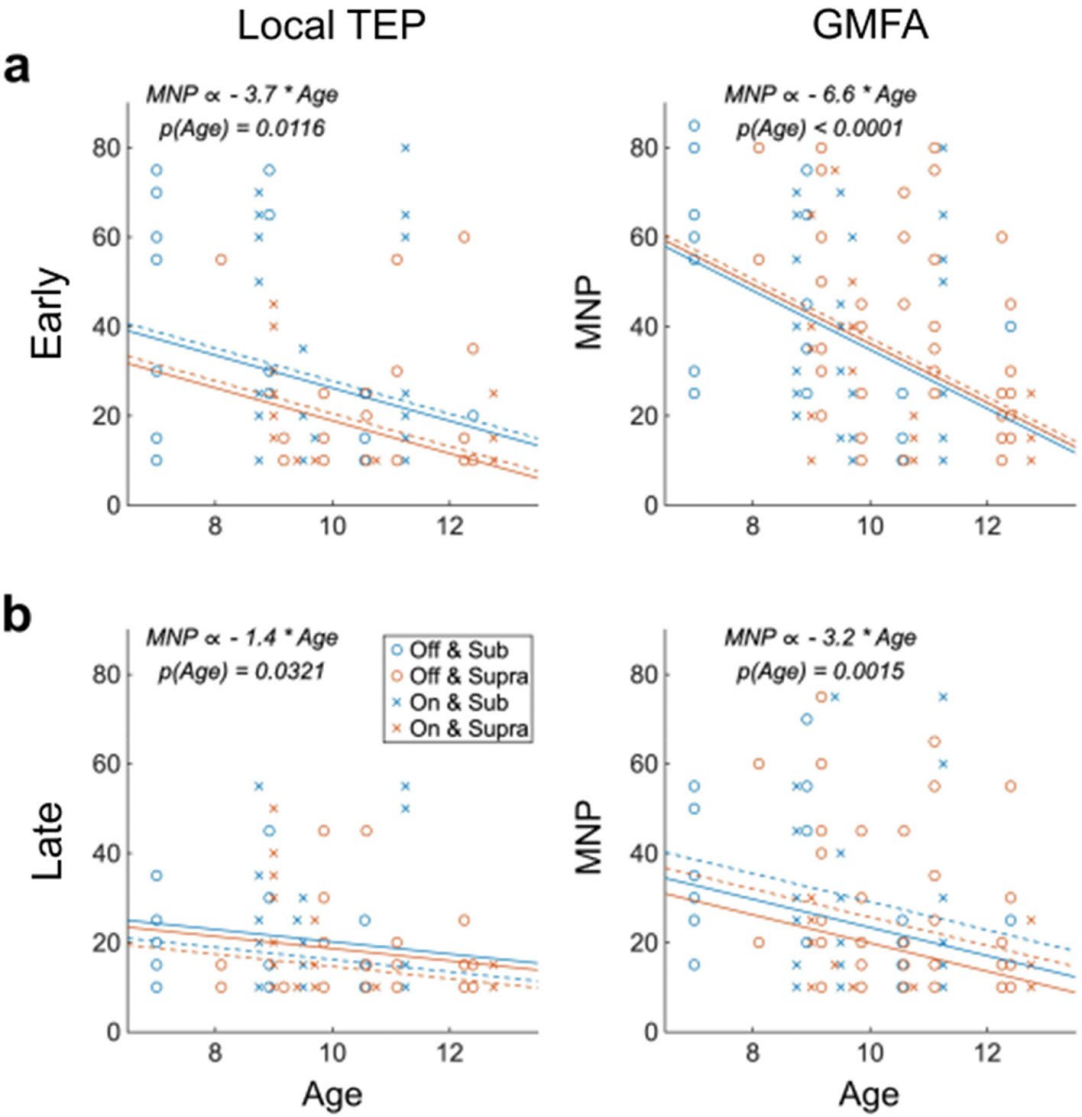
Multivariable models showing impact of three clinical factors on stability of the local TMS-evoked potential (TEP, left column) and the global mean field amplitude (GMFA, right column) for the (**a**) early (15–80 ms) and (**b**) late (80–350 ms) segments. Cross and circle markers represent the minimum number of pulses (MNP) for subjects on and off antiseizure medications (ASMs), respectively. Blue and red markers represent the MNP for those receiving subthreshold and suprathreshold stimulation intensity, respectively. Lines represent the relationship between age and stability for those on (solid) and off (dashed) ASMs, and for those receiving subthreshold (blue) and suprathreshold (red) stimulation intensity.

**Table 3:** Multivariable model estimating association between clinical factors and TEP stability.

	Model 1: estimate (CI)	Model 2: estimate (CI)	Model 3: estimate (CI)	Model 4: estimate (CI)
Local TEP at early waveform(15–80 ms)				
Intercept	69.5 (39.7, 99.4)	63.9 (36.6, 91.2)	29.6 (18.4, 40.9)	64.5 (36.1, 92.9)
Age (ΔMNP/year)	− 4.6 (− 7.2, − 2.1)*	− 3.7 (− 6.5, − 0.8)^#^		− 3.7 (− 6.5, − 0.8)^#^
ASM use	− 0.6 (− 10.6, 9.3)		− 2.5 (− 12.4, 7.4)	− 1.6 (− 10.3, 7.1)
Suprathreshold stim		− 7.1 (− 16.3, 2.2)	− 10.5 (− 19.8, − 1.2)^#^	− 7.3 (− 16.6, 2.0)
Local TEP at late waveform (80–350 ms)				
Intercept	30.7 (21.0, 40.5)	31.0 (16.5, 45.6)	17.0 (13.4, 20.7)	29.9 (18.9, 40.8)
Age (ΔMNP/year)	− 1.6 (− 2.4, − 0.7)*	− 1.3 (− 2.9, 0.3)^#^		− 1.4 (− 2.6, − 0.1)^#^
ASM use	4.3 (0.2, 8.4)		3.6 (− 1.3, 8.6)	4.0 (− 0.3, 8.3)
Suprathreshold stim		− 2.4 (− 7.6, 2.8)	− 3.1 (− 7.2, 1.0)	− 1.5 (− 6.0, 3.0)
GMFA at early waveform (15–80 ms)				
Intercept	101.3 (80.3, 122.2)	101.7 (77.0, 126.4)	39.2 (25.2, 53.3)	102.0 (78.3, 125.7)
Age (ΔMNP/year)	− 6.4 (− 8.5, − 4.3)*	− 6.6 (− 9.5, − 3.8)*		− 6.6 (− 9.5, − 3.7)*
ASM use	− 1.5 (− 11.8, 8.8)		− 2.5 (− 16.4, 11.4)	− 1.3 (− 12.2, 9.7)
Suprathreshold stim		1.6 (− 9.1, 12.3)	− 4.1 (− 16.4, 8.2)	1.3 (− 9.9, 12.6)
GMFA at late waveform (80–350 ms)				
Intercept	62.8 (44.7, 80.9)	59.4 (41.6, 77.2)	31.1 (21.7, 40.5)	60.8 (42.4, 79.2)
Age (ΔMNP/year)	− 3.6 (− 5.3, − 1.9)*	− 3.3 (− 5.1, − 1.5)*		− 3.2 (− 5.1, − 1.2)*
ASM use	− 5.1 (− 12.5, 2.3)		− 6.4 (− 14.4, 1.6)	− 5.8 (− 13.1, 1.5)
Suprathreshold stim		− 2.4 (− 10.0, 5.2)	− 6.8 (− 15.0, 1.3)	− 3.5 (− 11.5, 4.5)

Models 1–3 estimate the relationship between each variable and stability when controlling for one additional variable. Model 4 estimates the relationship between each variable and TEP stability while adjusting for the other two. *p-value < 0.01, ^#^*p*-value < 0.05. Δ: change of. ASM = Antiseizure medication. MNP = Minimum Number of Pulses; Stim = stimulation.

As age and ASM use affect rMT^14,47^, we assessed the interplay between these three variables. Multivariable analyses showed that age remained significantly associated with TEP stability (both local TEP and GMFA in both early and late segments), even after adjusting for ASM use and/or stimulation intensity relative to rMT. Older children consistently had more stable TEPs. ASM use did not significantly impact stability in any case. The association between stimulation intensity relative to rMT and stability was no longer significant after adjusting for age. We visualized the multivariable Model 4 in Table 3 as Fig. 5 to show the impact of three clinical factors on TEP stability. More detailed visualization of multivariable model #1–3 can be found in Appendix C.

### Comparison of MNP from two preprocessing pipelines (Appendix A)

Supplementary analyses show that the required MNP to achieve TEP stability did not significantly differ based on the pre-processing pipeline chosen (*p* > 0.05 for the early and late, local and global TEPs).

### Comparison of MNP from pulse inclusion methods (Appendix B)

Supplementary analyses show that the required MNP to achieve TEP stability did not significantly differ based on pulse inclusion methods for generating candidate TEPs (*p* > 0.05 for the early and late, local, and global TEPs).

## Discussion

A critical step in designing efficient TMS–EEG studies is determining how much data must be collected to measure a stable TEP waveform while balancing the experimental duration. Children are an understudied population who are known to have higher amplitude signals, but also potentially greater noise due to more limited cooperation. In this study, we investigated the MNP required to achieve stable TEP waveforms from the bilateral motor cortices in 18 children with SeLECTS, a common pediatric epilepsy syndrome. We quantified stability using the CCC, a measure capturing the similarity between two waveforms, and tested whether experimental or clinical factors affected stability of the TEP. We found that stable TEPs could be derived from fewer than 100 pulses, a number typically used in TMS–EEG experiments of healthy adults. We found that the later segment of the TEP was more stable than the earlier one and that the local TEP was more stable than the GMFA. Moreover, we found that older children had more stable TEPs than younger children, and TMS administered at an intensity above the rMT yielded more stable local TEPs than lower intensity stimulation.

TEPs are waveforms with peaks at different latencies reflecting different characteristics of neural responses^1,2^. The early segment reflects local cortical responses, while the later segment is linked to widespread network-level neural responses^35,36^. The later segment of the TEP is known to also reflect sensory evoked potentials^51^, even when careful experimental conditions are used to mask the auditory and somatosensory input from the TMS. Our results suggest that the early segment of TEP requires a greater number of pulses to achieve stability than the late segment. One possible explanation is that the early segment of TEPs may be influenced by immediate, but potentially more variable, neural responses, while the late segment might represent the convergence of neural response from multiple sources (e.g., somatosensory)^52^ rendering it less sensitive to transient fluctuations. For example, one previous study showed that TEPs at late latencies are site-invariant and more stable compared to early latencies^53^. Additionally, our results suggest that TEPs derived from local channels are more stable than the GMFA derived from all channels. A potential explanation is that local TEPs, derived from a smaller cortical region than GMFA, exhibit greater stability because they capture specific neural circuits with less functional and structural diversity^54^. In line with this, previous studies^17^ have demonstrated that global responses differed more than local responses after adjusting for stimulation parameters.

A number of experimental and biological factors^10,13,14,16^ affect TEP latency and amplitude and thus would be expected to affect TEP stability. Here, we explored the impact of several factors on stability, because TMS-EEG studies of pediatric clinical populations cannot account for factors like age or medication use simply through inclusion/exclusion criteria. Of the factors we investigated, age most consistently affected stability. Older children showed more stable local and global responses both in the early and late segments of the waveform. We had expected younger children to show greater TEP stability, because TEP, and particularly N100, amplitudes decreases with age in healthy children^24,55^, children with attention deficit hyperactivity disorder^56^ and children with epilepsy^23^. An intuitive explanation is that older children are better able to sit still and provide better “quality” data with fewer artifacts. However, this is unlikely to be the entire explanation as we did not see a strong relationship between age and the number of rejected channels, components’ variance, or trials (Fig. 4). A separate possibility is that younger children have greater variability in underlying brain signals, affecting the calculation of TEPs. This is supported by prior work finding that younger children have more variability in resting state EEG power^57,58^ as well as in visual and auditory evoked potentials^59^. Another consideration is the later components of TEPs, particularly after 80 ms^51^ overlap in time with sensory evoked potentials. While we made efforts to mask the somatosensory and auditory components of the TMS stimulation, children may still experience some of this sensory input. While accommodation to sensory input stabilizes by age 7 years^60^, the amplitude of sensory evoked potentials is larger in younger ages. Therefore, these artifacts (and the attenuation they undergo) may have a disproportionate impact on stability in younger vs. older children.

We next explored the impact of stimulation intensity on stability, comparing children for whom we were able to stimulate above rMT versus those for whom we were not able to stimulate at this intensity. The impact of stimulation intensity on stability needs to be carefully considered when designing experiments of children as they have higher rMT than adults, sometimes exceeding the maximal stimulator output^61^. Thus, suprathreshold stimulation may require using quite high intensity settings or may not be achievable for some pediatric participants. This is particularly a concern in patients with epilepsy as ASM use can also increase rMT^47^. Stimulating at higher intensity relative to rMT improves the SNR^10^, and we did indeed find that local TEPs were more stable in children stimulated at intensities exceeding rMT. The statistically significant effect of stimulation intensity on local TEP stability was lost after adjusting for age, which is not surprising as rMT and age are highly correlated in children^14^. Though stimulation intensity does not retain statistical significance after adjusting for age, it exerts the greatest independent effect on MNP of the early, local potentials. We therefore suggest that researchers consider adding extra pulses if a pediatric participant has a very high or unmeasurable rMT. In contrast to local TEP stability, GMFA stability did not significantly differ based on stimulation intensity relative to rMT. The GMFA, because it captures a broader range of channels, may be less sensitive to the effects of stimulation intensity on SNR compared to local TEPs.

We also found that stability of the early TEPs was higher on day 1 than day 2. One potential reason could be that the participants were more alert and attuned to the experiment on the first day as the stimulation was novel. This heightened alertness could lead to more consistent neural response and TEPs^62^.

We did not find ASM use had any significant effects on TEP stability. We tested the impact of ASMs as other studies^5,47,63,64^ have documented that ASMs can affect TEP amplitude and latency. The patients in our study treated with ASMs took either oxcarbazepine or levetiracetam. Voltage gated sodium channel blockers, like oxcarbazepine, raise rMT^47^. Levetiracetam may also increase rMT in healthy subjects, though its effects are less consistent across studies^65^. Given this, it might be expected that ASMs would reduce stability by increasing rMT and thus reducing the SNR. However, ASMs also have variable effects on the amplitude of specific TEP peaks. Single doses of carbamazepine (a voltage gated sodium channel blocker with a similar mechanism of action to oxcarbazepine) in healthy adults decrease the amplitude of P25 and P180 and increase the amplitude of N45 over the stimulated site^66^. Another study found that levetiracetam increases the amplitude of N45 while suppressing the amplitude of P70 on channels near the stimulated site^67^. Our stability analyses measured concordance of the entire waveform and thus may have missed peak-specific changes, though subdivision of the waveform into early and later periods at least partially addresses this. Our sample size of patients on specific medications was quite small, limiting our ability to fully investigate the effects of ASM use on stability. Importantly, we note that most TMS-neuropsychopharmacological studies investigate the impact of a single dose of these agents on healthy individuals, but we were studying their impact on children with epilepsy who were on stable doses of these medications chronically.

Finally, we sought to compare TEP stability in our pediatric sample with that reported in other populations. Prior studies^32,68,69^ in adults have discussed the “reliability” or “reproducibility” of the TEP, defined as whether peak amplitudes and latencies remained the same over varying degrees of time. Our definition of stability overlaps with the concept of reliability, focusing on the MNP required within a stimulation block to obtain a grand-average waveform that does not change with addition of extra pulses. Very few studies have explicitly investigated the stability of TEPs, though there is a general consensus across methodological papers that 100–200 pulses provide an adequate SNR in most brain regions^7–9^. One study^32^ measured TEP amplitudes and latencies at multiple regions after dorsolateral prefrontal cortex stimulation in 16 healthy adults. They compared TEPs measured within a stimulation block, across blocks within one day, and across days separated by 1 week, also using CCC. They found that fifty pulses were adequate to achieve a CCC > 0.8 within a stimulation block; this overlaps with our definition of stability and the MNP is similar to our findings. Additionally, they found that 60–100 pulses were optimal for achieving reliable TEPs across days. In line with our work, this group also found that early TEPs were less reliable than later N100 and P200 peaks. Another study^68^ focused on reliability only across days, stimulating two sites (left motor and left dorsolateral prefrontal cortex) on two days in seven healthy adults, with each stimulation block consisting of 100 pulses. They found that peak amplitudes and latencies were highly concordant across days (*r* > 0.8) both in the regions underlying the coil and in matched regions in the contralateral hemisphere. This study tested different stimulation intensities (90%, 100%, and 110% of rMT) and noted that lower intensities yielded smaller peaks but did not comment on the impact on reliability. Reliability across greater periods of time, such as across days is important when using TEPs as a biomarker for response to an intervention, where the outcome of interest is brain change due to that intervention. Stability within a block is also a crucial concept for guiding more efficient study designs through balancing data quality and experimental duration, particularly for studies investigating rapidly changing, time-sensitive brain states (e.g., a pre-seizure state) or in populations with lower tolerance for long studies (e.g., children, patients with specific medical conditions).

While our study highlighted that stable TEPs can be extracted from pediatric data, future work will also assess how best to interpret these evoked potentials. Several previously proposed methods^70–72^ explicitly account for noise autocorrelation and noise characteristics, which is particularly important in populations where SNR variability is more pronounced. Building upon this foundation, future research could explore more nuanced approaches to quantifying the impact of the SNR on TEP analyses, using methods like the standardized measurement error^73^. This may be especially relevant when working with diverse clinical populations or under experimental conditions where it is more challenging to control environmental or biological factors that contribute to noise.

### Limitations

Our study investigates TEP stability in a group of children with a specific epilepsy syndrome, and therefore the generalizability of this data to other pediatric groups is unclear. Historically, ethical restrictions have largely limited the conduct of TMS research on children without medical problems, but as increasing evidence^74^ supports the safety of TMS in this age group, we expect that institutional review boards may permit these studies moving forward. We therefore believe our work adds critical information for rigorous study design. Furthermore, children with SeLECTS by definition have normal macroscopic brain anatomy and a relatively mild, self-resolving form of epilepsy where seizures are rare and typically occur only during sleep. Therefore, information from this population is likely more generalizable than that gathered from children with more severe neurologic or psychiatric disorders and thus is a useful first step until we can gather data from children without medical problems.

A second limitation is that we simplified clinical factors, for example binarizing stimulation intensity and ASM use, and thus may be missing subtle effects of these factors. Larger studies could better explore these factors. A third limitation is that we were unable to identify rMT in some of our participants (i.e., rMT > 100% MSO), making it challenging to standardize stimulation intensity across all participants; despite this, all subjects had recognizable TEPs. Future studies could use E-field estimation or TEP amplitudes^10^ to standardize intensity across participants, including those with elevated rMT. Additionally, our analysis used the CCC to identify similarities in waveform shape, rather than amplitude or latency of specific peaks. We thought this was a more rigorous method as children have more simple waveforms than adults^75^, and there is less literature in this age group to define “normal” peaks; however, this is distinct from previous adult studies focused on peak measurements. A fourth limitation is that we assumed that stability could be reached within 100 pulses and therefore, by definition, every block reaches stability once all of the pulses are included. TEPs derived from high percentages of pulses (i.e. 80 of the 100 pulses) could thus falsely appear “stable” due to the diminishing influence of the remainder of the data. We believe this did not significantly impact our results, however, as stability was reached with only 30–50 pulses for the vast majority of our measurements. A fifth limitation is that we only used a single CCC threshold for determining stability. We chose 0.8 as the threshold of CCC based on prior literature^32,33^. The stringency of the stability threshold will vary depending on the purpose of a study, and studies attempting to identify small differences (e.g. between groups, due to treatment) may require even more stable measures. Finally, while the scope of this analysis was focused on TEP stability within-block at one stimulation site, future studies could look at stability of TEPs of other stimulation sites or the reliability of signal across time.

## Conclusion

Our data suggest that stable TEPs in children can be derived from less than 100 pulses per condition. When designing TMS-EEG studies for children, it is crucial to consider several key factors that influence stability. First, the age of the participants plays a significant role, with younger children typically needing additional pulses to achieve stable TEPs. Second, extra pulses may be needed for participants with elevated rMT, particularly in those whose rMT exceeds MSO. Finally, the signal of interest should be considered; additional pulses may be needed when investigating the early waveform or the global brain response. Tailoring study design to balance adequate data acquisition with feasible experimental duration will be critical for expanding TMS-EEG methods to pediatric populations.

### Supplementary Information

Supplementary Information.

## Data Availability

The data analyzed in this study was gathered as part of a larger clinical trial (NCT04325282) of TMS-EEG in SeLECTS. There are no reproduced materials. The datasets generated and/or analyzed during the current study are not publicly available due to privacy concerns. The data is clinical data that has not been deidentified. But the code used for the analysis is publicly available at https://github.com/Pediatric-Neurostimulation-Laboratory/TEP-Stability and the data is available from the corresponding author on reasonable request.

## Abbreviations

TMS–EEG: Transcranial magnetic stimulation paired with electroencephalography
TEP: TMS evoked potential
MNP: Minimum number of pulses
SeLECTS: Children with self-limited epilepsy with centrotemporal spikes
rMT: Resting motor threshold
MSO: Maximum stimulator output
SNR: Signal-to-noise ratio
ASM: Antiseizure medication
MRI: Magnetic resonance image
EMG: Electromyography
ABP: Abductor pollicis brevis
GMFA: Global mean field amplitude
CCC: Concordance correlation coefficient
GEE: Generalized estimating equation
