# Supplementary material for: Stability of transcranial magnetic stimulation electroencephalogram evoked potentials in pediatric epilepsy
Source: Sci Rep. 2024 Apr 20;14:9045. doi: 10.1038/s41598-024-59468-8 (PMC11031596; doi:10.1038/s41598-024-59468-8)
Supplement: Supplementary file 1 — Supplementary Information. [file 41598_2024_59468_MOESM1_ESM.docx]

**Supplementary Materials**

**Appendix A. Preprocessing pipeline comparison (Supplementary Figure 1-2)**

We conducted a comparative analysis between our TMS-EEG preprocessing pipeline with the TESA pipeline [^44,45^](https://www.zotero.org/google-docs/?qVCIrN), a common method for TMS-EEG data preprocessing semi- or fully-automatically. In this supplementary comparison, we used the fully-automatic version (i.e., without manual bad trial rejection). The primary goal of this analysis was to determine if stability (i.e., MNP) remained consistent when using different preprocessing pipelines. The secondary goal was to test the similarity of preprocessed TEP waveforms from two preprocessing pipelines and provide details (e.g., butterfly plots and topographies) of our preprocessed TEPs used in stability analyses.

**Data**: We picked 3 age-matched participants whose rMT was lower than the 100% MSO in both hemispheres. For each participant, we chose 4 blocks to cover all experimental factors (i.e, stimulated block order, hemisphere, and day): Block 1 and Block 2 were both left hemisphere stimulation on day 1, Block 3 was right hemisphere stimulation on day 1, and Block 4 was stimulated at the left hemisphere on day 2. As such, in a total of 12 blocks were preprocessed by two pipelines.

**Methodology of compared TESA pipeline**: Code for the TESA pipeline was adapted from “pipeline 1” described in [^45^](https://www.zotero.org/google-docs/?jTUyZ8). Data was epoched from -1 to 1.5 seconds around the pulse, and a baseline correction was applied by subtracting the mean between -500 to -10 milliseconds from all data points. Afterward, TMS pulse was removed (-2 to 12 millisecond). The first round of FastICA was performed on the data for identifying and removing decay artifacts using heuristic rules. Missing data were then interpolated using a cubic function (fit on 1 ms of data before and after the pulse), after which the data were downsampled to 1 kHz. The data were then band-pass (1-200), and band-stop (58-62 Hz) filtered using a zero-phase finite response filter. The second round of FastICA was performed to further remove artifacts representing eye blinks, lateral eye movement and persistent muscle activity using heuristic rules. Finally, data were re-referenced to a common average. Filtering parameters (e.g., low-, high-, and band-pass filtering) and selected timespan for epoching and baseline correction were kept consistent between TESA and our pipeline.

**Comparison of stability between pipelines (supplementary figure 1)**: we calculated the required MNP to reach stability of 96 waveforms (early/late * local/GMFA * 4 blocks * 3 participants * 2 pipelines) using the same criteria described in the Methods. We then compared these MNPs at either early or late segments of either local TEP or GMFA between two pipelines using paired t-tests (in a total of 4 paired t-tests). Results show that the required MNP to achieve stability is not significantly different between pipelines (Supplementary Figure 2, p > 0.05 in all 4 tests). Specifically, the MNP from our pipeline at the *early* segment of *local TEP* was 19 +/- 12, while the corresponding MNP from the TESA pipeline was 19 +/- 13. The MNP from our pipeline at the *late* segment of *local TEP* was 23 +/- 14, while the corresponding MNP from the TESA pipeline was 21 +/- 13. The MNP from our pipeline at the *early* segment of *GMFA* was 44 +/- 25, while the corresponding MNP from the TESA pipeline was 43 +/- 24. The MNP from our pipeline at the *late* segment of *GMFA* was 29 +/- 20, while the corresponding MNP from the TESA pipeline was 30 +/- 19. Such results are matched with our expectation because we believe the goal of preprocessing steps is to separate out cortical response from artifacts. As long as the gold-standard TEP and candidate TEPs were yielded from the same pipeline, the required MNP to achieve stability remains similar across pipelines.

**Comparison of TEP waveforms between pipelines (supplementary figure 2)**: We next compared early and late segments of local TEPs and GMFA of each block yield from two pipelines using the CCC. Results show that two pipelines yielded similar TEPs and GMFA waveforms (90%, 43/48, of comparisons had a CCC > 0.8). There were dissimilarities between the two TEPs at early waveforms, which may result from the different preprocessing algorithms used between our pipeline and the TESA pipeline. For example, the way of interpolating the missing data after the TMS pulse removal and bandpass filtering differs between pipelines; the primary method of handling the decay artifacts also differs (our pipeline added a dedicated fitting & smoothing strategy [^40^](https://www.zotero.org/google-docs/?18E76K), while the TESA pipeline relies on the first stage of ICA for this); our primary pipeline uses the SOUND algorithm, but our tested TESA pipeline does not. Previous studies have demonstrated that variations in pipelines, even with a single-step difference, can substantially influence the final TEPs [^45^](https://www.zotero.org/google-docs/?HMP6j4). Presently, there is an absence of a universally validated “optimal” pipeline for analyzing TMS-EEG data. It is important to tailor the pipeline steps to align with the specific characteristics of the data and the research focus. In light of this, we would like to emphasize that the observed dissimilarities in the early segment of preprocessed TEP waveforms did not exert a significant impact on stability, which is the primary focus of this study.


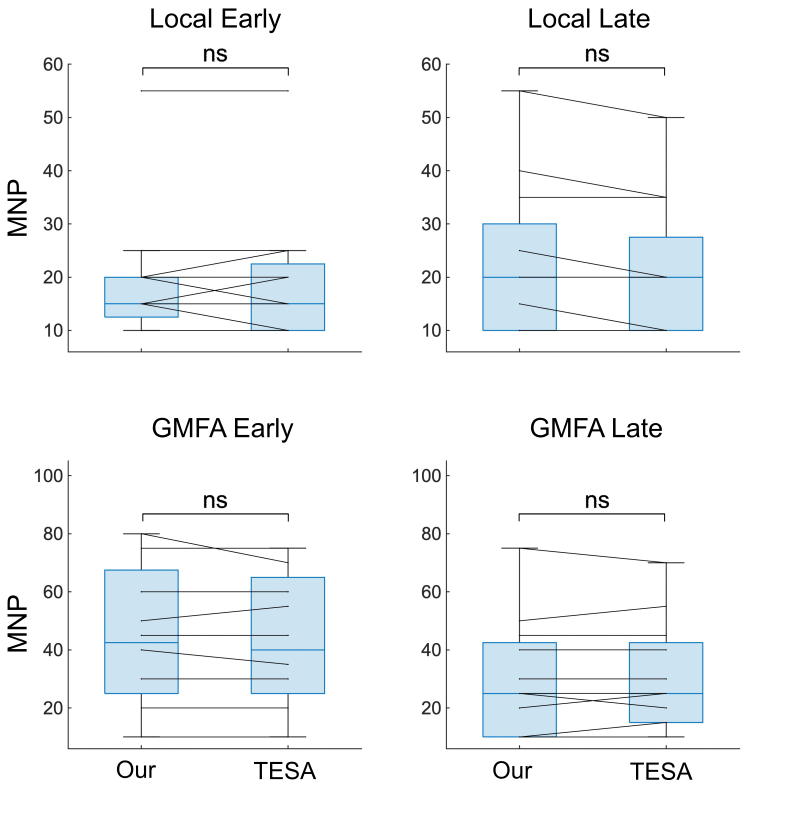


Supplementary Figure 1: Comparison of the required minimum number of pulses (MNP) to achieve TEP stability using data preprocessed by two pipelines. Black line connects the same block processed by two pipelines. Top: local TEP stability; bottom: GMFA stability. Left: stability at early waveforms; right: stability at late waveforms. No significant difference exists in any paired t-test of four measurements. Note that points with the same value of MNP are overlapped on the figures.


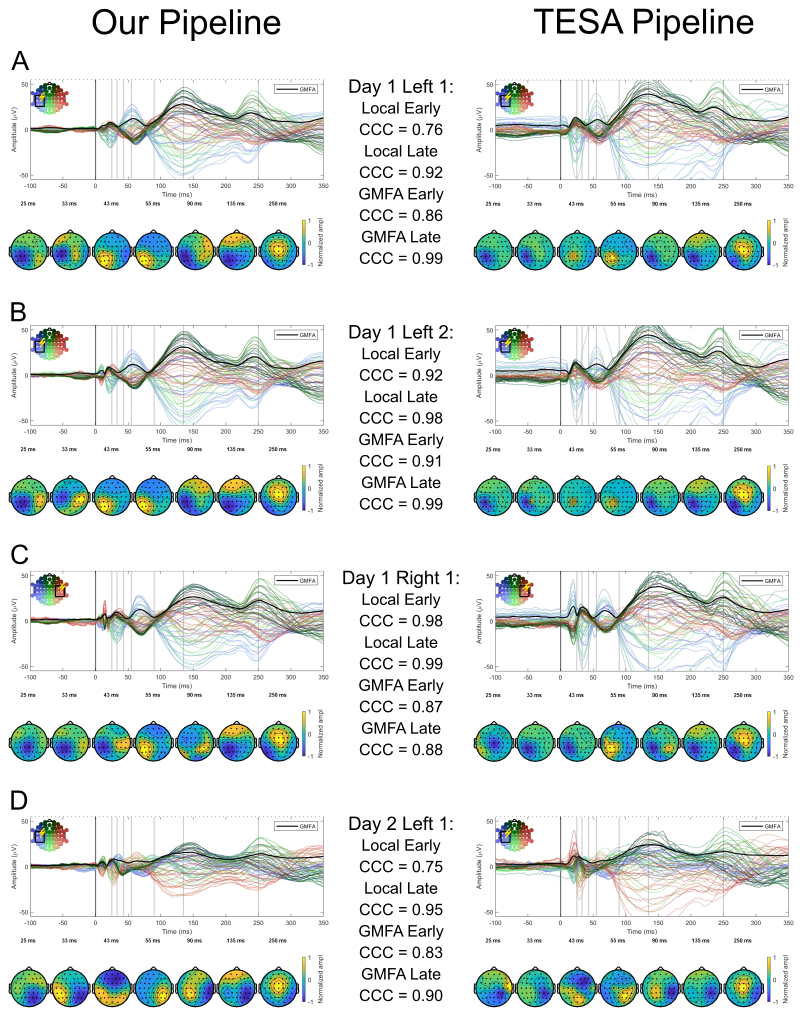


Supplementary Figure 2: Comparison of preprocessing pipeline between our pipeline (left) with TESA pipeline (right) at different blocks (A to B), stimulated sites (A to C) and days (A to D) using data from one exemplary participant. Black solid line on each plot represents the GMFA averaged from all channels. Black circles with a lighting symbol mark out the stimulated site. Color of stimulated channels are matched with color lines in the butterfly plots. Bottom panel of each subplot shows the topographic of brain excitability at 25, 33, 43, 55, 90, 135, and 250 milliseconds to the TMS onset.

**Appendix B. Analysis on pulse inclusion randomization (Supplementary Figure 3)**

To test whether entrainment affected the MNP, we compared our original method for generating “candidate” waveforms to a method in which pulses included were chosen at random from the stimulation block.

**Data**: We included two blocks of data for each of the 18 participants (one left and one right hemisphere, both obtained on the same day), thus including 36 blocks in this supplementary analysis.

**Methods**: This method deviated from our original consecutive pulse inclusion strategy, where we started with 10 pulses and progressively added 5 consecutive pulses. Instead of adding sequential pulses, here we chose pulses from the block by randomly adding them to the candidate average waveform without replacement (choosing only among pulses not already incorporated into the average). We continued this process iteratively, ensuring that each new candidate TEP included a randomly chosen subset of pulses, progressively increasing in number. This approach aimed to minimize any potential order effects or biases from consecutive pulse inclusion.

The MNPs calculated for each of the 36 blocks in this analysis were compared to the MNP from the original analysis using the consecutive pulse inclusion method with a paired t-test to determine if there was a significant difference in the MNPs derived from the two methods.

**Results**: The paired t-test revealed that there was no significant difference in the MNPs calculated using the two methods (Supplementary Figure 5, p > 0.05 in all 4 tests). Specifically, when comparing the consecutive vs. random method, there was no significant difference in the MNP of the early, local TEP (19 +/- 15 vs. 17 +/- 14, p=0.287); the late, local TEP (14 +/- 8 vs. 14 +/- 10, p = 0.720); the early GMFA (35 +/- 22 vs. 33 +/- 20, p = 0.109), or the late GMFA (23 +/- 18 vs. 21 +/- 17, p = 0.067).

This suggests that the original consecutive method of pulse inclusion does not introduce a significant bias in the calculation of MNPs in our cohort. Nevertheless, in studies with high risk of entrainment or pulse influencing, a randomized pulse inclusion method can better help with validation of stability analyses.


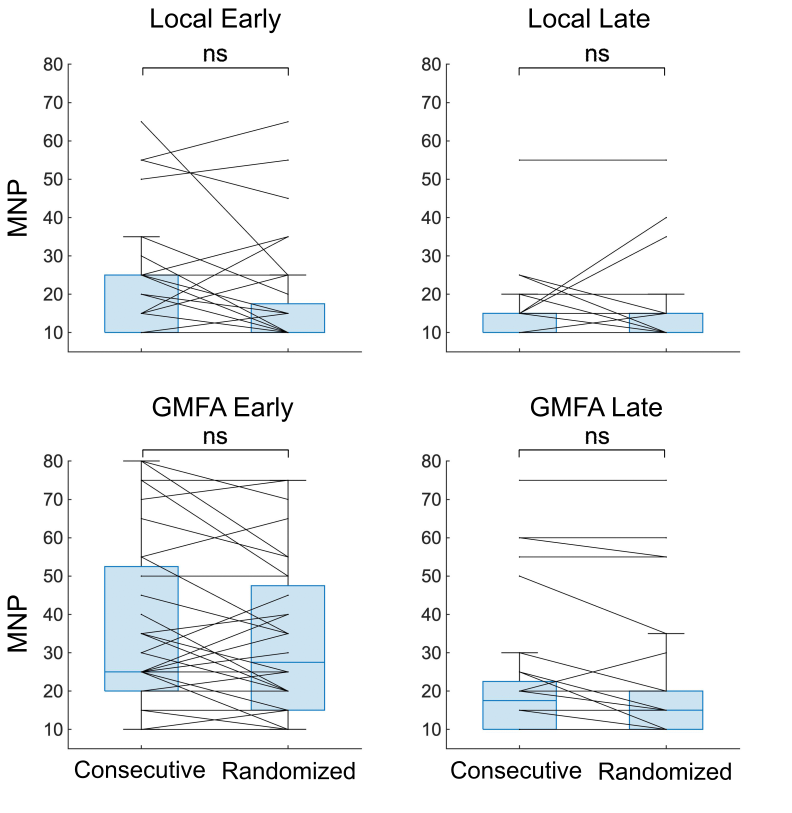


Supplementary Figure 3: Comparison of the required minimum number of pulses (MNP) to achieve TEP stability using epochs added in consecutive vs. randomized order. Black line connects the MNP yield from the same block using two methods. Top: local TEP stability; bottom: GMFA stability. Left: stability at early waveforms; right: stability at late waveforms. No significant difference exists in any paired t-test of four measurements. Note that points with the same value of MNP are overlapped on the figures.

**Appendix B. Multivariable model comparisons (Supplementary Figure 4-5)**

**
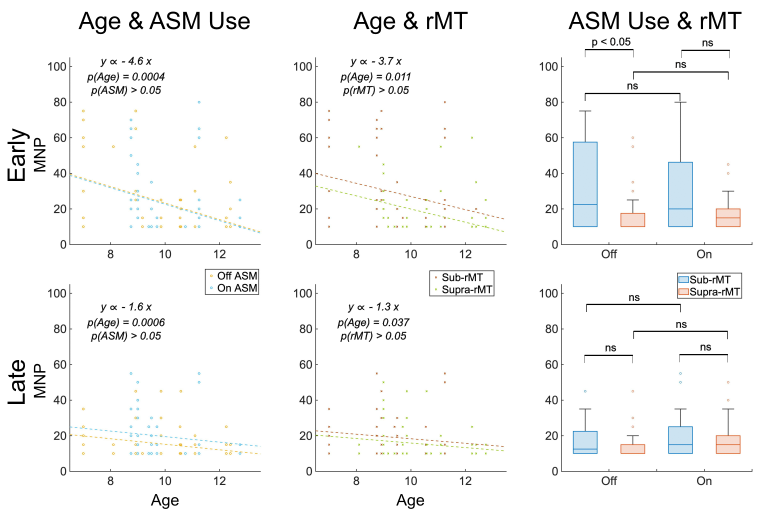
**

Supplementary Figure 4: Multivariable models showing impact of three clinical factors on the **local** TEP stability for the early (15–80ms; top), and late (80-350ms; bottom) waveform. Left: Relationship between age and stability for those on (blue) and off (yellow) ASM use. Center: Relationship between age and stability for those receiving subthreshold (red) and suprathreshold (green) stimulation intensity. Right: Relationship between ASM use and stability for those receiving subthreshold (blue) and suprathreshold (red) stimulation intensity.

**
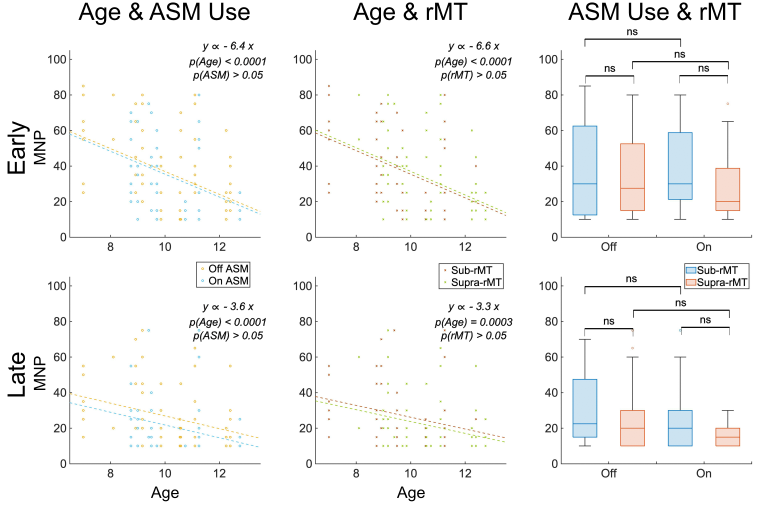
**

Supplementary Figure 5: Multivariable models showing impact of three clinical factors on the **GMFA** stability for the early (15–80ms; top), and late (80-350ms; bottom) waveform. Left: Relationship between age and stability for those on (blue) and off (yellow) ASM use. Center: Relationship between age and stability for those receiving subthreshold (red) and suprathreshold (green) stimulation intensity. Right: Relationship between ASM use and stability for those receiving subthreshold (blue) and suprathreshold (red) stimulation intensity.
